# New records of Limoniidae and Pediciidae (Diptera) from Croatia

**DOI:** 10.3897/zookeys.513.10066

**Published:** 2015-07-15

**Authors:** Levente-Péter Kolcsár, Marija Ivković, Ivančica Ternjej

**Affiliations:** 1 Hungarian Department of Biology and Ecology, Babeș-Bolyai University, Clinicilor 5-7, 400006 Cluj-Napoca, Romania; 2 Department of Zoology, Division of Biology, Faculty of Science, University of Zagreb, Rooseveltov trg 6, 10000 Zagreb, Croatia

**Keywords:** *Lipsothrix*, *Paradelphomyia*, *Rhabdomastix* and *Tricyphona*, Plitvice Lakes, Krka River, distribution

## Abstract

New records are provided for Limoniidae and Pediciidae from Croatia, with new distribution records for species in 12 different genera. Four genera and 18 species are newly recorded for Croatia. Until now, including this data, 87 Limoniidae and eight Pediciidae have been recorded from Croatia. In this paper we confirm presence of Ormosia (Oreophila) bergrothi (Strobl, 1895) and we give the first records of Dicranota (Paradicranota) pavida (Haliday, 1833) and Molophilus (Molophilus) repentinus Starý, 1971 from Balkan Peninsula.

## Introduction

Pediciidae or hairy-eyed crane flies comprise slender tipuloid like flies. Species belonging to subfamily Pediciinae have carnivorous larvae and are connected with the wet environments (Kolcsár et al. 2012). The adults can be found near the larval habitats (brooks, springs, small rivers), and they are mostly found in riparian vegetation. Most of the species are mountainous. In some species males can produce massive swarms (e.g. Pedicia (Amalopis) occulta (Meigen, 1830), Dicranota (Paradicranota) pavida (Haliday, 1833)). Limoniidae or short-palped crane flies are a paraphyletic group (Petersen et al. 2010) of various sized tipuloid like flies, which larvae occupy different habitats (Ujvárosi 2005, Ujvárosi and Póti 2006, Kolcsár et al. 2013). A large part of genera bind to wet environments and some are truly aquatic (e.g. *Antocha*). From Western Palaearctic region 77 Pediciidae and 735 Limoniidae species are reported (Oosterbroek 2015).

The Croatian Pediciidae and Limoniidae fauna is less studied, despite the relatively large number of publications. The first study which contains records and description of *Dactylolabis dilatata* from Croatia was noted by Loew (1856). Since then Egger (1863), Strobl (1893, 1900, 1902), Langhoffer (1917), Lackschewitz (1928, 1940a, 1940b), Lackschewitz and Pagast (1941, 1942), Coe (1959), Nielsen (1959), Simova (1960), Mannheims (1967), Starý (1969, 1971a), Mendl (1984) and Savchenko et al. (1992) contributed additional distribution records from Croatia. Oosterbroek and Simova-Tosic (2004) gave a list of Pediciidae and Limoniidae species of Croatia. In the latest publication by Starý and Oosterbroek (2008) Dicranomyia (Glochina) tristis (Schummel, 1829) is reported for the first time in Croatia. At the present a total of four Pediciidae and 73 Limoniidae are reported from Croatia (Oosterbroek 2015). In this paper we offer new records of several genera of Pediciidae and Limoniidae collected from various sites in Croatia.

## Material and methods

In the course of various ecological and taxonomic projects and surveys by Marija Ivković, many limonid and pedicid flies were collected by means of emergence traps set in springs, tufa barriers, streams and small rivers at nine sites in Plitvice Lakes National Park and at two sites at Krka National Park. Traps were emptied once a month, at the end of each month. Each trap had a surface area of 45 × 45 cm (and height 50 cm), was fixed in the sediment of the stream, and contained 2% formaldehyde; six traps were placed at each location on various types of microhabitats. At majority of sites traps were placed for one year (two sites at Krka River) or two years (majority of sites in Plitvice Lakes), but at three sites at Plitvice Lakes (spring of Bijela rijeka, tufa barrier Labudovac and tufa barrier Kozjak-Milanovac) they were placed for seven years, for additional details see Ivković et al. (2014). Each trap was recorded with the initial “P” and a number, e.g. “P4", is pyramid emergence trap no. 4. All flies were collected from March 2007 to October 2014.

Additional sampling using a sweep net took place between March 2011 and June 2014 at various sites in Croatia. All Limoniidae and Pediciidae specimens were placed in 80% ethanol. The literature used for identification is as follows: Alexander (1975), Blythe (2010), Dienske (1987), Podenas et al. (2006), Savchenko (1982), Starý (1971a, 1971b, 2004, 2009), Starý and Rozkošný (1970), Tjeder (1958, 1972), Ujvárosi and Bálint (2012) and Stary and Stubbs (2015). All the material listed here is deposited in the col. M. Ivković, University of Zagreb, Croatia (UZC) and Diptera Collection of the Faculty of Biology and Geology, Cluj-Napoca, Romania. GPS coordinates and altitudes for the localities where specimens were trapped and/or collected are given in Table 1.

**Table 1.** The list of sampling sites.

Site name	Longitude	Latitude	Altitude (m)
Spring Jankovac, Papuk Mountain	E 17°41'14"	N 45°31'06"	525
Dubočanka stream, Papuk Mountain	E 17°40'42"	N 45°29'11’’	585
Brzaja, before N. Zvečeva, Papuk Mountain	E 17°30'53"	N 45°33'17"	502
Brzaja after N. Zvečeva, Papuk Mountain	E 17°31'53"	N 45°30'57"	368
Spring of Bijela rijeka, Plitvice Lakes	E 15°33'43"	N 44°50'05’’	720
Upper reach of Bijela rijeka, Plitvice Lakes	E 15°33'33"	N 44°50'04’’	715
Spring of Crna rijeka, Plitvice Lakes	E 15°36'28"	N 44°50'14"	680
Crna rijeka by the bridge, Plitvice Lakes	E 15°35'59"	N 44°50'22"	665
Tufa barrier Labudovac, Plitvice Lakes	E 15°35'59"	N 44°52'17’’	630
Tufa barrier Kozjak-Milanovac, Plitvice Lakes	E 15°36'32"	N 44°53'39’’	545
Tufa barrier Novakovića Brod, Plitvice Lakes	E 15°36'38"	N 44°54'07"	500
Stream Plitvica,Plitvice Lakes	E 15°36'27"	N 44°54'08"	555
Korana village, Plitvice Lakes	E 15°37'09"	N 44°55'33’’	390
Spring of Krupa River	E 15°54'33"	N 44°11'49"	150
Spring Krčić	E 16°19'42"	N 44°01'48’’	390
Spring of Krka River	E 16°14'07"	N 44°02'31’’	265
Roški Slap, Krka River	E 15°58'22"	N 43°54'20’’	55
Skradinski Buk, Krka River	E 15°57'55"	N 43°48'09’’	45
Radmanove Mlinice, Cetina River	E 16°45'11"	N 43°26'16"	15

## Results

### Faunistic records

The following format is used for the records given here: name of the site, followed by the sampling date (in the case of collections from the pyramid emergence traps, the trap number is also given, e.g. “P1" is pyramid emergence trap number 1), and the number of sampled specimens. New species for Croatia are listed with * before the name of the species. All the sites are listed in Table 1. All mentioned distribution data are from Catalogue of the Craneflies of the World (Oosterbroek 2015).

### Pediciidae

#### Subfamily Pediciinae

* **Dicranota (Dicranota) bimaculata (Schummel, 1829)**

**New records. Spring of Bijela rijeka, Plitvice Lakes**, 5.x.2010, P4, 1♀; same site, 29.v.2012, P3, 1♀

**Comments.** Widespread in Europe except the Mediterranean region. In the Balkan Peninsula it has been recorded from Bulgaria.

* **Dicranota (Paradicranota) pavida (Haliday, 1833)**

**New records. Upper reach of Bijele rijeka, Plitvice Lakes**, 5.x.2010, P4, 1♂, 1♀; **spring of Crna rijeka, Plitvice Lakes**, 29.v.2011, 1♂

**Comments.** Mostly Central and Western European distributed species. This is the first record for Balkan Peninsula.

* **Pedicia (Amalopis) occulta (Meigen, 1830)**

**New records. Spring of Bijela rijeka, Plitvice Lakes**, 28.iv.2007, P2, 1♀; same site and date, P3, 1♂; same site and date, P6, 1♀; same site, 29.v.2007, P3, 1♂, 2♀; same site, 28.vi.2007, P2, 1♀; same site and date, P5, 1♂; same site, 25.vii.2007, P2, 1♂; same site, 30.viii.2007, P2, 1♂; same site and date, P3, 1♂; same site, 2.x.2007, P3, 1♂; same site, 29.x.2007, P2, 3♂; same site and date, P3, 2♂, 3♀; same site and date, P5, 1♀; same site, 29.xi.2007, P2, 1♀; same site and date, P3, 1♀; same site, 26.xii.2007, P3, 2♂; same site, 2.iv.2008, P2, 1♂, 1♀; same site and date, P6, 1♂; same site, 23.iv.2008, P2, 1♂, 1♀; same site and date, P3, 1♂; same site and date, P4, 1♀; same site and date, P6, 1♂; same site, 1.vi.2008, P2, 1♀; same site and date, P4, 2♀; same site, 30.vi.2008, P3, 1♂; same site, 22.vii.2008, P4, 1♀; same site, 27.viii.2008, P4, 1♂; same site and date, P6, 1♀; same site, 26.xi.2008, P2, 1♂; same site and date, P3, 1♀; same site, 3.iii.2009, P2, 1♂; same site, 1.iv.2009, P2, 2♂, 2♀; same site and date, P4, 1♂; same site and date, P6, 1♀; same site, 2.v.2009, P2, 2♂; same site and date, P6, 1♀; same site, 30.x.2009, P2, 1♂, 1♀; same site, 8.iv.2010, P2, 1♀; same site and date, P3, 1♂; same site, 4.v.2010, P6, 1♀; same site, 4.vi.2010, P4, 1♂; same site, 25.vii.2010, P2, 1♂, 1♀; same site, 26.viii.2010, P4, 2♂; same site, 2.ix.2010, P3, 1♀; same site and date, P4, 1♀; same site and date, P6, 1♀; same site, 10.xii.2010, P3, 1♂, 1♀; same site, 25.ii.2011, P6, 1♂, 1♀; same site, 26.iv.2011, P4, 2♀; same site and date, P6, 1♂, 1♀; same site, 29.v.2011, P4, 1♂, 2♀; same site and date, P6, 1♂; same site, 27.vi.2011, P2, 1♂; same site, 2.viii.2011, P6, 1♂, 1♀; same site, 6.ix.2011, P3, 1♀; same site and date, P4, 2♀; same site, 4.viii.2012, P3, 1♂; same site, 4.ix.2012, P3, 1♂; same site and date, P4, 2♂, 1♀; same site, 6.xi.2012, P4, 1♂; same site and date, P5, 1♂, 1♀; same site and date, P6, 1♂; same site, 11.iii.2013, P2, 2♂; same site, 2.iv.2013, P3, 1♀; same site and date, P4, 1♀; same site, 3.v.2013, P4, 1♂; same site, 7.viii.2013, P4, 1♀; same site and date, P5, 1♂; same site, 29.x.2013, P3, 1♀; **upper reach of Bijele rijeka, Plitvice Lakes**, 30.iii.2007, P2, 1♀; same site and date, P4, 2♂; same site, 28.iv.2007, P2, 1♀; same site and date, P3, 2♂; same site and date, P4, 2♂; same site and date, P5, 2♀; same site, 29.v.2007, P2, 2♂; same site and date, P3, 1♀; same site and date, P4, 1♀; same site and date, P5, 1♂; same site and date, P6, 1♀; same site, 28.vi.2007, P2, 1♂, 2♀; same site and date, P3, 1♂; same site and date, P4, 1♂; same site, 25.vii.2007, P3, 2♀; same site and date, P4, 6♂, 1♀; same site, 30.viii.2007, P1, 1♀; same site and date, P2, 3♂; same site and date, P3, 1♂; same site and date, P4, 1♂; same site, 2.x.2007, P1, 2♂, 1♀; same site and date, P2, 2♀; same site and date, P3, 1♂, 1♀; same site and date, P4, 1♀; same site and date, P6, 1♂; same site, 29.x.2007, P1, 2♀; same site and date, P5, 1♀; same site, 29.xi.2007, P1, 1♀; same site and date, P2, 1♂, 3♀; same site, 25.ii.2008, P1, 1♂; same site, 2.iv.2008, P2, 1♂; same site and date, P3, 4♂; same site and date, P4, 2♂, 3♀; same site, 23.iv.2008, P2, 1♂, 1♀; same site and date, P4, 3♂; same site and date, P5, 2♀; same site, 1.vi.2008, P5, 1♀; same site, 30.vi.2008, P2, 2♂; same site and date, P3, 1♂, 1♀; same site and date, P4, 1♂; same site, 22.vii.2008, P3, 1♂; same site, 27.8.2008, P1, 1♀; same site and date, P2, 1♂; same site and date, P3, 1♂; same site and date, P5, 1♀; same site, 29.xi.2008, P6, 1♀; same site and date, 27.x.2008, P2, 2♀; same site and date, P3, 1♂; same site and date, P4, 1♂; same site and date, P6, 1♀; same site and date, 26.xi.2008, P2, 1♀; same site and date, P4, 1♀; same site, 3.iii.2009, P5, 1♂; same site, 1.iv.2009, P3, 1♀; same site and date, P5, 1♂; same site, 2.v.2009, P4, 1♂, 1♀; same site and date, P5, 1♂, 1♀; same site, 29.v.2009, P1, 2♀; same site and date, P2, 1♂; same site and date, P3, 1♂; same site, 26.vi.2009, P4, 1♀; same site and date, P5, 1♀; same site, 24.vii.2009, P4, 1♀; same site and date, P5, 1♀; same site, 27.viii.2009, P3, 1♂; same site and date, P4, 1♂; same site, 30.x.2009, P2, 3♂, 2♀; same site, 8.iv.2010, P6, 1♂; same site, 4.v.2010, P2, 1♂, 2♀; same site and date, P4, 1♀; same site, 1.vii.2010, P1, 1♂; same site and date, P4, 1♀; same site and date, P6, 1♂; same site, 25.vii.2010, P1, 2♂; same site and date, P2, 1♂; same site and date, P4, 6♀; same site and date, P6, 1♂; same site, 26.viii.2010, P2, 2♂; same site and date, P4, 2♂, 3♀; same site and date, P6, 1♂; same site, 5.x.2010, P2, 2♂, 1♀; same site and date, P3, 1♂, 1♀; same site and date, P4, 1♂; same site, 2.xi.2010, P1, 4♀; same site and date, P3, 1♂; same site and date, P4, 1♂, 3♀; same site and date, P5, 1♀; same site, 10.xii.2010, P1, 1♂; **spring of Crna rijeka, Plitvice Lakes**, 29.v.2007, P3, 1♂; same site, 30.viii.2007, P5, 1♀; **Crna rijeka by the bridge**, 28.vi.2007, P3, 1♂;

**Comments.** Widespread in Europe, including the Balkan Peninsula. It is frequently collected in springs and headwater brooks (Ujvárosi and Bálint, 2012). In the Balkan Peninsula it is recorded from Albania, Bosnia and Herzegovina, Bulgaria, Greece, Macedonia, Montenegro, Serbia and Slovenia.

* **Tricyphona (Tricyphona) immaculata (Meigen, 1804)**

**New records. Spring of Bijela rijeka, Plitvice Lakes**, 28.x.2011, P6, 1♂; **upper reach of Bijele rijeka, Plitvice Lakes**, 29.v.2007, P4, 2♀; **spring of Crna rijeka, Plitvice Lakes**, 30.iv.2007, P1, 5♀; same site, 29.v.2007, P1, 5♀; **tufa barrier Labudovac, Plitvice Lakes**, 28.x.2011, P1, 1♂

**Comments.** Widespread species. In the Balkan Peninsula it is recorded from Bosnia and Herzegovina, Montenegro, Macedonia, Serbia and Slovenia.

### Limoniidae

#### Subfamily Chioneinae

* **Ellipteroides (Ellipteroides) lateralis (Macquart, 1835)**

**New record. Tufa barrier Kozjak-Milanovac, Plitvice Lakes**, 1.vii.2012, P4, 1♂

**Comments.** The species is frequently collected in calcareous springs, fens and around the tufa sediment (Blythe 2010). It is widely distributed all around Europe.

**Gonomyia (Gonomyia) tenella (Meigen, 1818)**

**New records. Spring of Bijela rijeka, Plitvice Lakes**, 29.x.2007, P5, 1♀; **Korana Village, Plitvice Lakes**, 29.vi.2007, P2, 1♀; same site and date, P6, 1♀; same site, 26.vii.2007, P1, 6♀; same site and date, P2, 1♂; same site and date, P3, 1♂; same site and date, P6, 3♂, 12♀; same site, 1.xi.2007, P1, 1♀; same site and date, P6, 3♀

**Comments.** A semi-aquatic species which can be found along flowing and standing waters (Kolcsár et al. 2013). In the Balkans it is recorded from Bulgaria, Croatia, Macedonia and Slovenia.

* **Molophilus (Molophilus) brevihamatus Bangerter, 1947**

**New record. Spring of Krka River**, 7.vii.2011, 1♂

**Comments.** A characteristic species along mountainous waters in Carpathians, Alps and Balkan high mountains (Pirin, Rila). Probably the microclimate of the spring provides right condition for the species in lower altitude. In the Balkan Peninsula it is recorded from Bulgaria and Slovenia.

* **Molophilus (Molophilus) bifidus Goetghebuer, 1920**

**New record. Spring of Bijela rijeka, Plitvice Lakes**, 4.9.2012, P1, 1♂

**Comments.** This species is associated with springs and head waters (Ujvárosi 2005). Previous records from the Balkan Peninsula are from Bosnia and Herzegovina, Bulgaria and Macedonia.

**Molophilus (Molophilus) obscurus (Meigen, 1818)**

**New records. Spring Krčić**, 29.iv.2011, 2♂; **Roški Slap, Krka River**, 13.x.2011, 1♂

**Comments.** Common species, with wide distribution in Europe, including Balkan Peninsula.

* **Molophilus (Molophilus) repentinus Starý, 1971**

**New record. Korana Village, Plitvice Lakes**, 30.v.2007, P2, 1♂, 1♀

**Comments.** Mountains species with sporadic distribution in Alps and Carpathians and surrounding mountains area. Its presence in the low altitude (390 m) is explainable by the presence of mountainous microhabitats around fast flowing Korana River. First record for Balkan Peninsula.

* **Ormosia (Oreophila) bergrothi (Strobl, 1895)**

**New record. Spring of Crna rijeka, Plitvice Lakes**, 25.vii.2007, P1, 3♀; same site, 30.viii.2007, P1, 1♀

**Comments.** This species has a sporadic distribution, with erroneous remark on type- locality. Oosterbroek and Simova-Tosic (2004) suggested that the type-locality is not in Croatia and removed the species from Croatian checklist. This is the first confirmation of this species present in Croatia and Balkan Peninsula.

* **Rhabdomastix (Rhabdomastix) edwardsi Tjeder, 1967**

**New records. Korana Village, Plitvice Lakes**, 29.vi.2007, P2, 1♂; same site, 29.ix.2008, P6, 1♀

**Comments.** Riverine species associated with sandy sediments mostly in mountainous areas (Boardman 2012). Reported from Albania, Austria, Bosnia and Herzegovina, Bulgaria, Czech Republic, France, Germany, Great Britain, Italy, Slovakia, Slovenia and Spain.

* ***Rhypholophus phryganopterus* Kolenati, 1860**

**New record. Spring of Crna rijeka, Plitvice Lakes**, 30.iv.2007, P1, 1♂

**Comments.** Montane species, distributed in Alps, Carpathians and Bulgarian mountains.

#### Subfamily Limnophilinae

***Eloeophila apicata* (Loew, 1871)**

**New record. Upper reach of Bijele rijeka, Plitvice Lakes**, 25.vii.2007, P4, 1♀

**Comments.** Common species associated with sandy sediment (Ujvárosi 2005).

***Eloeophila maculata* (Meigen, 1804)**

**New records. Upper reach of Bijele rijeka, Plitvice Lakes**, 2.x.2007, P5, 1♀; **Crna rijeka by the bridge, Plitvice Lakes**, 31.viii.2007, P2, 1♀

**Comments.** Common species with wide European distribution.

* ***Eloeophila miliaria* (Egger, 1863)**

**New record. Brzaja, before N. Zvečeva, Papuk Mountain**, 14.vi.2012, 1♀

**Comments.** Less common species than *Eloeophila apicata* and *Eloeophila maculata*. Reported from Albania, Bulgaria and Serbia.

**Epiphragma (Epiphragma) ocellare (Linnaeus, 1760)**

**New records. Spring of Kupa River**, 10.vi.2009, 1♀; **spring of Crna rijeka, Plitvice Lakes**, 29.v.2011, 1♂

**Comments.** Very common species with wide distribution.

**Hexatoma (Eriocera) chirothecata (Scopoli, 1763)**

**New records. Tufa barrier Novakovića Brod, Plitvice Lakes**, 25.vii.2007, P4, 1♂; **Korana Village, Plitvice Lakes**, 26.vii.2007, P4, 1♀

**Comments.** Mostly distributed in southeastern Europe. Reported from majority of Balkan countries.

* **Paradelphomyia (Oxyrhiza) senilis (Haliday, 1833)**

**New records. Upper reach of Bijele rijeka, Plitvice Lakes**, 2.x.2007, P2, 1♂; same site and date, P4, 1♀; **spring of Crna rijeka, Plitvice Lakes**, 28.vi.2007, P1, 2♀

**Comments.** A wide distributed species in Palearctic region, frequently collected around different flowing waters (Ujvárosi 2005, Kolcsár et al. 2013). In the Balkan Peninsula it is recorded from Bulgaria, Greece and Macedonia.

#### Subfamily Limoniinae

**Antocha (Antocha) vitripennis (Meigen, 1830)**

**New record. Tufa barrier Kozjak-Milanovac, Plitvice Lakes**, 27.vi.2013, P4, 1♂; **tufa barrier Novakovića Brod, Plitvice Lakes**, 1.vi.2008, P2, 1♂; **Korana Village, Plitvice Lakes**, 30.v.2007, P6, 1♂; same site and date, 29.vi.2007, P1, 1♂; same site and date, 30.vi.2008, P6, 1♂; **Radmanove mlinice, Cetina River**, 19.vii.2005, 1♂, 5♀.

**Comments.** A very common species.

**Dicranomyia (Dicranomyia) chorea (Meigen, 1818)**

**New records. Spring of Bijela rijeka, Plitvice Lakes**, 1.vii.2010, P6, 1♂; same site, 2.viii.2011, P6, 1♂; **upper reach of Bijele rijeka, Plitvice Lakes**, 27.viii.2009, P3, 2♀; 1.vii.2010, P3, 1♂; same site, 25.vii.2010, P3, 1♂; same site, 26.viii.2010, P1, 1♂, 1♀; same site and date, P3, 4♂, 2♀; same site and date, P4, 2♂; same site, 5.x.2010, P1, 1♀; same site and date, P2, 1♂; **tufa barrier Kozjak-Milanovac, Plitvice Lakes**, 29.x.2013, P1, 1♂; **stream Plitvica, Plitvice Lakes**, 30.viii.2007, P3, 1♂; **Korana village, Plitvice Lakes**, 29.vi.2007, P1, 1♂; **Roški Slap, Krka River**, 28.iv.2014, P4, 1♀;

**Comments.** A very common species.

**Dicranomyia (Dicranomyia) didyma (Meigen, 1804)**

**New records. Spring of Bijela rijeka, Plitvice Lakes**, 30.viii.2007, P5, 15♂, 9♀; same site and date, P6, 6♂, 5♀; same site, 2.x.2007, P2, 1♂, 2♀; same site and date, P5, 4♂, 2♀; same site, 29.x.2007, P5, 1♂; same site, 1.vi.2008, P5, 1♀; same site, 22.vii.2008, P6, 4♂; same site, 27.viii.2008, P6, 1♀; same site, 29.ix.2008, P5, 1♀; same site and date, P6, 2♂; same site, 1.x.2009, P5, 1♂; same site and date, P6, 3♂, 3♀; same site, 30.x.2009, P5, 1♂, 2♀; same site, 8.xii.2009, P6, 1♂, 1♀; same site, 1.vii.2010, P6, 1♀; same site, 26.viii.2010, P5, 4♂, 8♀; same site and date, P6, 1♂; same site, 5.x.2010, P2, 1♂, 1♀; same site and date, P5, 4♂, 3♀; same site and date, P6, 2♂, 2♀; same site, 29.v.2011, P6, 7♂, 6♀; same site, 2.viii.2011, P6, 2♂, 1♀; same site, 6.ix.2011, P6, 12♂, 10♀; same site, 28.ix.2011, P6, 2♀; same site, 3.ix.2013, P6, 7♂, 5♀; **upper reach of Bijele rijeka, Plitvice Lakes**, 24.vii.2009, P1, 1♂; same site and date, P3, 1♂; same site, 27.viii.2009, P1, 5♂, 1♀; same site and date, P2, 1♀; same site, 1.x.2009, P1, 8♂, 2♀; same site and date, P2, 2♀; same site and date, P3, 3♂, 4♀; same site, 30.x.2009, P2, 1♂; same site and date, P3, 1♀; same site, 1.vii.2010, P2, 4♂, 4♀; same site and date, P3, 1♂; same site, 25.vii.2010, P2, 4♂, 5♀; same site, 26.viii.2010, P1, 3♂, 3♀; same site and date, P3, 1♂, 3♀; same site and date, P4, 2♂; same site, 5.x.2010, P1, 1♂, 2♀; same site and date, P2, 2♂; same site and date, P3, 14♂; same site and date, P4, 1♂, 3♀; same site, 2.xi.2010, P3, 3♀; same site and date, P4, 1♂, 8♀; same site, 10.xii.2010, P4, 1♀; **tufa barrier Kozjak-Milanovac, Plitvice Lakes**, 1.vi.2008, P4, 1♂; same site, 27.viii.2008, P5, 1♀; same site, 1.vii.2010, P4, 1♂, 4♀; **stream Plitvica, Plitvice Lakes**, 25.v.2007, 1♂; same site, 27.vii.2007, P2, 1♂; **Roški Slap, Krka River**, 28.iv.2011, 3♂, 2♀; same site, 25.v.2011, 5♂, 3♀; same site, 30.viii.2011, 1♀; same site, 13.x.2011, 1 ♂; same site, 6.xi.2013, P4, 1♂, 1♀; same site, 7.ii.2014, P4, 5♂, 1♀; same site, 5.iii.2014, P4, 1♀; same site, 2.iv.2014, P3, 1♂; same site and date, P4, 1♂, 4♀; same site, 28.iv.2014, P4, 2♂, 3♀; same site, 2.vi.2014, P3, 1♂, 2♀; same site and date, P4, 3♂, 2♀; same site, 26.vi.2014, P3, 2♂, 1♀; same site and date, P4, 5♂, 1♀; same site, 26.vii.2014, P4, 1♂, 1♀; same site and date, P6, 2♂; **Skradinski Buk, Krka River**, 2.vi.2014, P3, 3♂, 2♀; same site, 26.vi.2014, P4, 1♀

**Comments.** A very common and abundant species.

**Dicranomyia (Dicranomyia) goritiensis (Mik, 1864)**

**New records. Roški Slap, Krka River**, 28.iv.2011, 2♂; same site, 25.v.2011, 2♂; same site, 2.vi.2014, P4, 1♀; same site, 26.vii.2014, P2, 1♂; same site and date, P4, 1♂, 2♀; same site, 2.ix.2014, P4, 1♀; same site, 2.x.2014, P1, 2♀; same site, 27.x.2014, P2, 2♀; same site and date, P4, 2♂; **spring of Krka River**, 25.v.2011, 1♂; **spring of Krupa River**, 26.v.2011, 1♂

**Comments.** Sporadic distributed species, which is associated with moss and/or algae covered stones, mostly around waterfalls and rocky coast. In the Balkan Peninsula it is recorded from Croatia and Greece.

* **Dicranomyia (Dicranomyia) imbecilla Lackschewitz, 1941**

**New records. Spring of Bijela rijeka, Plitvice Lakes**, 29.v.2011, P2, 1♀; same site, 27.vi.2013, P1, 1♀; **tufa barrier Kozjak-Milanovac, Plitvice Lakes**, 27.vi.2013, P5, 1♂; **spring Krčić**, 29.iv.2011, 1♂; same site, 26.v.2011, 3♂, 4♀

**Comments.** The species is known from calcareous seepages depositing tufaceous substrate. The species known from Bulgaria, Czech Republic, Great Britain, Germany, Slovakia and Switzerland (Stary and Stubbs 2015).

* **Dicranomyia (Dicranomyia) lucida de Meijere, 1918**

**New record. Roški Slap, Krka River**, 2.x.2014, P1, 1♀

**Comments.** Relatively common species in woodlands around rivers. In the Balkan Peninsula it is reported from Bulgaria, Macedonia and Greece.

**Dicranomyia (Dicranomyia) mitis (Meigen, 1830) complex**

**New records. Brzaja after N. Zvečevo, Papuk Mountain**, 13.vi.2012, 1♂; **upper reach of Bijele rijeka, Plitvice Lakes**, 29.v.2009, P1, 1♂;; **spring of Krka River**, 7.vii.2011, 1♀

**Comments.** A very common and widespread species.

**Dicranomyia (Dicranomyia) modesta (Meigen, 1818)**

**New record. Roški Slap, Krka River**, 25.v.2011, 2♂

**Comments.** A common species which inhabits various habitats, but mostly found in forests.

* ***Elliptera omissa* Schiner, 1863**

**New record. Dubočanka stream, Papuk Mountain**, 18.ix.2012, 1♀

**Comments.** Mountainous species. In the Balkan Peninsula it is reported from Bosnia and Herzegovina, Bulgaria, Montenegro, Serbia and Slovenia.

***Limonia hercegovinae* (Strobl, 1898)**

**New records. Upper reach of Bijele rijeka, Plitvice Lakes**, 28.iv.2007, P4, 1♂; **spring of Crna rijeka, Plitvice Lakes**, 30.iv.2007, P1, 1♂, 1♀; **spring Jankovac, Papuk Mountain**, 13.vi.2012, 1♀

**Comments.** A widely distributed species, but mostly in Balkan Peninsula.

***Limonia macrostigma* (Schummel, 1829)**

**New record. Brzaja, after N. Zvečeva, Papuk Mountain**, 13.vi.2012, 1♂

**Comments.** Widely distributed and common species.

***Limonia phragmitidis* (Schrank, 1781)**

**New record. Brzaja, after N. Zvečeva, Papuk Mountain**, 14.vi.2012, 2♀

**Comments.** Widely distributed and common species.

* ***Lipsothrix nobilis* Loew, 1873**

**New record. Crna rijeka by the bridge, Plitvice Lakes**, 28.vi.2007, P3, 1♂; **tufa barrier Labudovac, Plitvice Lakes**, 31.v.2007, P2, 2♂

**Comments.** Less common species associated with woodland streams and springs.

* ***Lipsothrix remota* (Walker, 1848)**

**New records. Roški Slap, Krka River**, 28.iv.2011, 1♂; same site, 25.v.2011, 1♂, 1♀

**Comments.** Less common species associated with woodland streams and springs.

**Orimarga (Orimarga) virgo (Zetterstedt, 1851)**

**New record. Spring of Krka River**, 7.vii.2011, 2♀

**Comments.** Species reported from the different types of calcareous rocky habitats, which are covered with moss and/or algae. In the Balkan Peninsula it is reported from Croatia, Greece and Slovenia.

## Discussion

In this paper we report for the first time four genera (*Lipsothrix* Loew, *Paradelphomyia* Alexander, *Rhabdomastix* Skuse and *Tricyphona* Zetterstedt) and 18 species new for Croatia. Including our results, eight Pediciidae and 87 Limoniidae have now been recorded from Croatia.

Oosterbroek and Simova-Tosic (2004) summarized the literature data of Former Yugoslav states, in which they reported 72 limonid and four pedicid species from Croatia and additional limonid species is reported by Starý and Oosterbroek (2008). This number is only the fragment of the real species number, which is supported by our results. Tipuloidea fauna is better studied in some neighboring countries, Slovenia has 11 species of Pediciidae (P) and 106 of Limoniidae (L), while Hungary has 12 species of Pediciidae and 132 of Limoniidae, however a similar number of species was reported from Bosnia and Herzegovina (6 P and 69 L), Serbia (8 P and 68 L) and Montenegro (9 P and 59 L). It is evident from these numbers that much remains to be discovered about the Tipuloidea fauna of Croatia and entire Balkan Peninsula.
